# Isoliquiritigenin Reverses Epithelial-Mesenchymal Transition Through Modulation of the TGF-β/Smad Signaling Pathway in Endometrial Cancer

**DOI:** 10.3390/cancers13061236

**Published:** 2021-03-11

**Authors:** Hsin-Yuan Chen, Yi-Fen Chiang, Jia-Syuan Huang, Tsui-Chin Huang, Yin-Hwa Shih, Kai-Lee Wang, Mohamed Ali, Yong-Han Hong, Tzong-Ming Shieh, Shih-Min Hsia

**Affiliations:** 1 School of Nutrition and Health Sciences, College of Nutrition, Taipei Medical University, Taipei 11031, Taiwan; hsin246@gmail.com (H.-Y.C.); yvonne840828@gmail.com (Y.-F.C.); m507104022@tmu.edu.tw (J.-S.H.); 2 Department of Nutrition, I-Shou University, Kaohsiung 84001, Taiwan; yonghan@isu.edu.tw; 3 Graduate Institute of Cancer Biology and Drug Discovery, College of Medical Science and Technology, Taipei Medical University, Taipei 11031, Taiwan; tsuichin@gmail.com; 4 Department of Healthcare Administration, Asia University, Taichung 41354, Taiwan; evashih@asia.edu.tw; 5 Department of Nursing, Ching Kuo Institute of Management and Health, Keelung 20301, Taiwan; kellywang@tmu.edu.tw; 6 Clinical Pharmacy Department, Faculty of Pharmacy, Ain Shams University, Cairo 11566, Egypt; mohamed.aboouf@pharma.asu.edu.eg; 7 School of Dentistry, College of Dentistry, China Medical University, Taichung 40402, Taiwan; tmshieh@mail.cmu.edu.tw; 8 School of Food and Safety, Taipei Medical University, Taipei 11031, Taiwan; 9 Nutrition Research Center, Taipei Medical University Hospital, Taipei 11031, Taiwan; 10 Graduate Institute of Metabolism and Obesity Sciences, College of Nutrition, Taipei Medical University, Taipei 11031, Taiwan

**Keywords:** endometrial cancer, isoliquiritigenin, epithelial–mesenchymal transition, transforming growth factor beta, metastasis

## Abstract

**Simple Summary:**

The high recurrence risk and poor prognosis of metastatic endometrial cancer are the main focus of interventional therapy. In view of this, we established in vitro and in vivo metastasis models and explored the underlying mechanisms of the epithelial-mesenchymal transition (EMT) process, cell migration ability, and metastasis in response to isoliquiritigenin (ISL). The presented in vitro and in vivo preclinical studies both demonstrated that ISL efficiently suppressed endometrial cancer cell migration and reduced the HEC-1A-LUC tumor metastasis in nude mice through inhibiting TGF-β/Smad signaling pathway. These findings shed the light for further research to highlight the ISL potential in endometrial cancer metastasis.

**Abstract:**

Endometrial cancer is a common gynecological cancer with a poor prognosis, mostly attributed to tumor metastasis. Epithelial–mesenchymal transition (EMT) can be mediated via transforming growth factor beta (TGF-β) signaling pathway, facilitating the ability of cancer cell invasion and migration. Isoliquiritigenin (ISL) is a flavonoid derived from licorice with reported antineoplastic activities. This study aims to investigate the anti-metastatic potential of ISL on endometrial cancer both in vitro and in vivo. First, human endometrial cancer cell lines (HEC-1A, Ishikawa, and RL95-2) were treated with ISL and then subjected to functional assays such as migration assay as well as molecular analyses including immunoblotting, immunofluorescence and RT-qPCR. In addition, HEC-1A-LUC cells were implanted into female nude mice and treated with ISL by intraperitoneal injection for four weeks. Results showed that ISL inhibited cell migration and reversed the effect of TGF-β on the expression of E-cadherin, N-cadherin, vimentin, α-SMA, p-Smad3, and TWIST1/2 In vitro. Interestingly, In vivo study revealed that ISL reduced peritoneal dissemination and serum level of TGF-β1, as well as decreased the expression levels of N-cadherin, p-Smad2/3, TWIST1/2, while increased E-cadherin. Overall, ISL reverses the EMT through targeting the TGF-β/Smad signaling pathway and features a potential therapeutic treatment for metastatic endometrial cancer.

## 1. Introduction

Endometrial cancer is the most common gynecological malignancy in women from Western countries which usually occurs later in life at menopause [1]. According to the American Cancer Society, there will be about 66,570 newly diagnosed cases in the United States in 2021, and mortality of about 12,940 women [2]. Unfortunately, up to 30% of tumors are invasive and gradually metastasize to extrauterine tissue, which lead to a higher risk of recurrence and poor prognosis [3,4,5]. Therefore, early diagnosis of endometrial cancer as well as slowing tumor metastasis are essential approaches for better patients’ prognosis.

In cancer pathophysiology, epithelial-mesenchymal transition (EMT) plays an important mechanism in mediating metastasis. More specifically, EMT is a process that allows epithelial cells to transition into mesenchymal cells, promoting tumor progression [6]. A previous study has shown that the status of EMT affects the prognosis of patients with endometrial cancer [7]. Importantly, loss of E-cadherin expression in the epithelial cells results in losing cell adhesion and cellular polarity, therefore considered a hallmark of EMT process. Moreover, decreased E-cadherin expression is coupled with increased mesenchymal N-cadherin expression level which in turn enhance cells ability to migrate and invade as shown in several studies [8,9,10]. Other mesenchymal markers in EMT include vimentin, fibronectin and α-smooth muscle actin (α-SMA), which are also markers for myofibroblast [11].

Transforming growth factor-β (TGF-β) is a multifunctional cytokine [12], which regulate cell proliferation and induce EMT, invasion, and angiogenesis, thus promoting cancer progression [13,14,15]. Particularly, TGF-β has a dual role during carcinogenesis. At earlier stage, TGF-β induces cell growth arrest and promotes apoptosis, while in advanced stage, TGF-β overexpression promotes tumor metastasis [16,17,18]. Examples include prostate, breast, ovarian, and endometrial tumors, where studies have shown that overexpression of TGF-β is associated with tumor metastasis and poor prognosis [15,19]. Briefly, TGF-β ligand binds to TGF-β receptors (type I and II), then Smad2 and Smad3 become phosphorylated, form a complex with Smad4, and this complex translocates into the nucleus, binding to DNA and regulating transcription of its downstream target genes such as repressing the expression of E-cadherin and inducing mesenchymal N-cadherin expression as well as degrading extracellular matrix (ECM), which collectively lead to cancer metastasis [20]. Recently, an attractive strategy for cancer treatment is to target Smad-independent TGF-β signaling pathway.

In recent years, studies exploring the effect of natural extracts on cancer chemoprevention are increasing due to their favored profile of low toxicity and high tolerance in humans [21]. Dietary flavonoids are among those compounds with potent effect on cancer progression, presumably via reducing DNA damage [22]. Lately, studies explored the relationship between flavonoids and metastasis with promising findings that flavonoids can delay the onset of cancer invasiveness and reduce the metastatic spread in several models [23].

Isoliqueritigenin (ISL) is a flavonoid derived from licorice and bean sprouts [24]. Studies reported that ISL has several functions, such as antioxidant [25], anti-inflammatory [26], and possesses antitumor activities [27]. Over the past decade, large number of studies explored the role of ISL in targeting pathways and molecular mechanisms involved in multiple cancers pathogenesis [28]. Most studies pointed a beneficial impact of ISL in cancer treatment including our previous study, which has shown that ISL can induce apoptosis, autophagy, and cell growth inhibition in human endometrial cancer [29] and ovary cancer [30]. In addition, ISL showed an inhibitory effect against benign tumors, such as uterine leiomyoma [31]. However, literature is lacking information regarding the potential influence of ISL on EMT and the TGF-β signaling pathway in endometrial cancer. Hence, the present study aims to investigate whether ISL has anti-metastatic effects on human endometrial carcinoma via inhibition of TGF-β signaling pathway both in vitro and in vivo.

## 2. Results

### 2.1. The Effect of Various Concentrations of ISL on Human Endometrial Cancer Cells and Human Endometrial Stromal Cells

**Figure 1:**
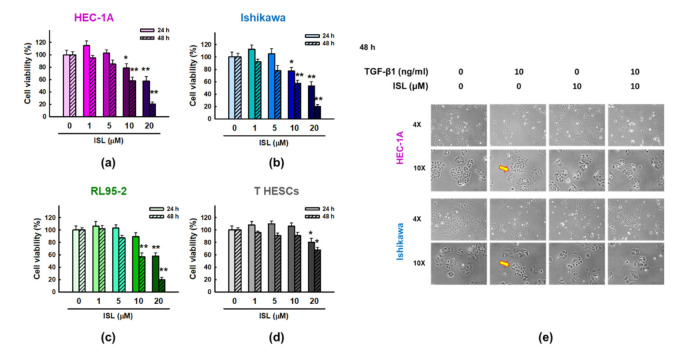
The effect of ISL at various concentrations on human endometrial cancer cells and human endometrial stromal cells. HEC-1A (**a**), Ishikawa (**b**), RL95-2 (**c**), and T HESCs (**d**) cells were treated with ISL (1, 5, 10, and 20 μM) for 24 h and 48 h. The cell viability was measured using the MTT assay. (**e**) Representative phase–contrast images showing the morphological changes of HEC-1A cells and Ishikawa human endometrial cancer cells treated without or with 10 ng/mL of TGF-β1 and 10 μM of ISL for 48 h.Images were captured at 400× magnification. The yellow arrow indicated the location of cells with spindle shape. The values were expressed as the mean ± SD of three independent experiments. * *p* < 0.05, ** *p* < 0.01 compared to the vehicle control.

Human endometrial cancer cell lines (HEC-1A, Ishikawa, and RL95-2) and human endometrial stromal T HESCs cells were treated with different concentrations of ISL (0, 1, 5, 10, and 20 μM), and cell viability was measured after 24 and 48 h of treatment using MTT assay. The results showed that treatment with ISL at concentrations 10 μM and 20 μM significantly reduced the survival rate of HEC-1A, Ishikawa, and RL95-2 cell lines compared with untreated control group (*p* < 0.01, Figure 1a–c). Interestingly, only 20 μM of ISL treatment slightly inhibited, yet statistically significant, the cell viability of normal T-HESCs cells (Figure 1d). These interesting findings reflect relative selective growth inhibitory effect of ISL, at concentration less than 20 μM, on cancer cells while not affecting normal endometrial stromal cells. Therefore, we selected these low doses of ISL (1, 5 and 10 μM) for subsequent experiments. In addition, since HEC-1A and Ishikawa endometrial cancer cells represent different clinical stages, we used both in the subsequent experiments.

Next, HEC-1A and Ishikawa cells were stimulated with 10 ng/mL of TGF-β1 for 48 h, and their morphology was observed and photographed by microscopy. The result showed that following TGF-β1 treatment, the cells acquired a mesenchymal like phenotype with a characterized spindle shape (the yellow arrow) (Figure 1e), reflecting their tendency to migrate. However, this phenomenon was abrogated after co-treatment with 10 μM ISL where HEC-1A and Ishikawa cells gradually restored their epithelial architecture (Figure 1e).

### 2.2. ISL Inhibits TGF-β1-Induced Migration of Human Endometrial Cancer Cells

**Figure 2:**
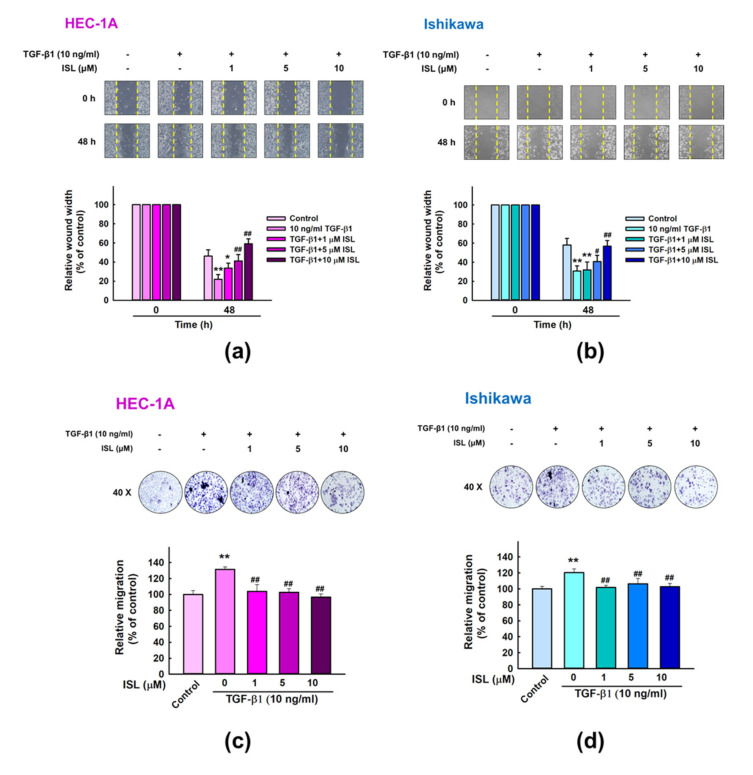
ISL inhibits TGF-β1-induced migration of human endometrial cancer cells. (**a**,**b**) The motility response of HEC-1A and Ishikawa cells to the administration of TGF-β1 (10 ng/mL) and ISL (1, 5, and 10 μM) were measured using the wound-healing assay. Images were captured at 0 and 48 h (100× magnification). (**c**,**d**) ISL (1, 5, and 10 μM) inhibits TGF-β1-induced migration of HEC-1A and Ishikawa cells, as confirmed by the transwell migration assay. Images were captured at 48 h (400× magnification). The quantitative values were expressed as the mean ± SD. * *p* < 0.05, ** *p* < 0.01 compared to the vehicle control. # *p* < 0.05, ## *p* < 0.01 compared to the TGF-β1 alone group.

Wound-healing and transwell migration assays were utilized to examine the effect of ISL and TGF-β1 on the migration ability of human endometrial cancer cells. After 48 h exposure, data showed that different concentrations of ISL significantly blocked TGF-β1-induced migration of HEC-1A and Ishikawa cells, and delayed the cell entry into the wound area in a dose dependent manner, as compared to the TGF-β1 (10 ng/mL) alone (*p* < 0.01, Figure 2a,b), with ISL 10 μM showing the most potent effect. Similar results were found using the transwell migration assay, where ISL significantly abrogated TGF-β1 induced cell migration through the transwell membrane and decreased the number of migrated cells (*p* < 0.01, Figure 2c,d). Taken together, ISL suppresses the migratory capacities of HEC-1A and Ishikawa cells mediated by TGF-β.

### 2.3. ISL Inhibits TGF-β1-Induced Expression of EMT Markers on Human Endometrial Cancer Cells

**Figure 3:**
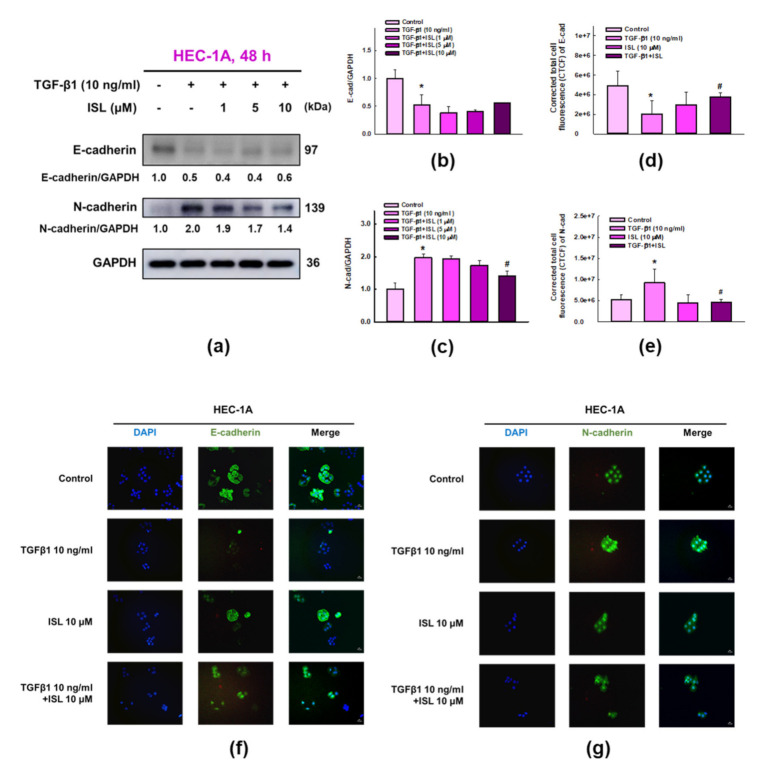
ISL reversed TGF-β1-induced epithelial-mesenchymal transition (EMT) in human endometrial cancer HEC-1A cells. Cells were treated with 10 ng/mL of TGF-β1 and ISL (1, 5, and 10 μM) for 48 h. Cell lysates were separated by SDS-PAGE and subjected to western blotting with the anti-E-cadherin, and N-cadherin (**a**). Each target protein was normalized to GAPDH and protein quantification was shown in bar graph (**b**,**c**). Immunofluorescence staining in HEC-1A cells were monitored in the presence or absence of TGF-β1 (10 ng/mL) and ISL (10 μM). Green color represents the staining of E-cadherin (**f**) or N-cadherin (**g**). Blue color represents nuclear DNA staining by DAPI (600× magnification). The corrected total cell fluorescence (CTCF) of E-cadherin (**d**) and N-cadherin (**e**) were quantified by ImageJ software. The quantitative values were expressed as the mean ± SD (*n* = 3). * *p* < 0.05, versus the vehicle control. # *p* < 0.05, versus the TGF-β1 alone group.

**Figure 4:**
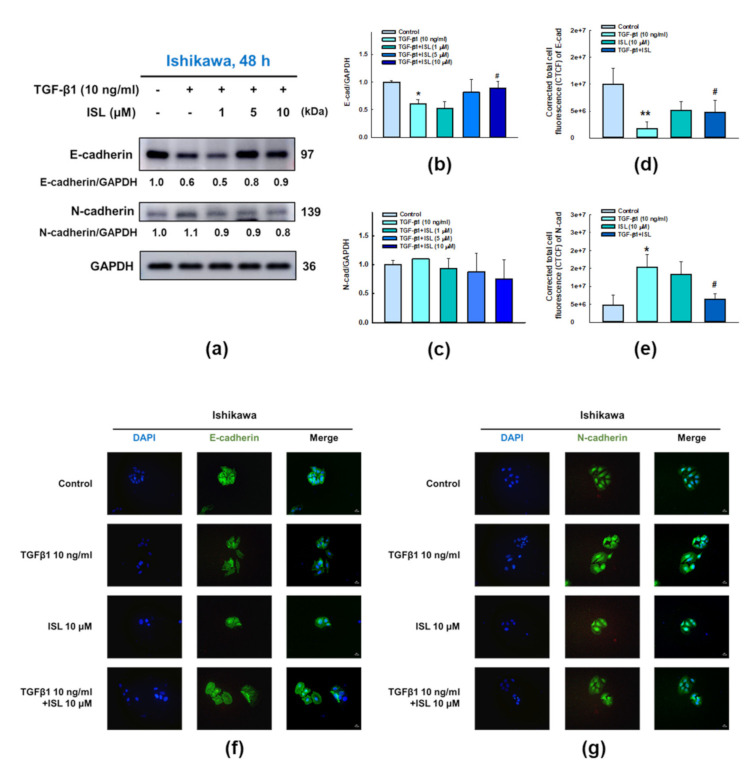
ISL reversed TGF-β1-induced epithelial-mesenchymal transition (EMT) in human endometrial cancer Ishikawa cells. Cells were treated with 10 ng/mL of TGF-β1 and ISL (1, 5, and 10 μM) for 48 h. Cell lysates were separated by SDS-PAGE and subjected to western blotting with the anti-E-cadherin, and N-cadherin (**a**). Each target protein was normalized to GAPDH and protein quantification was shown in bar graph (**b**,**c**). Immunofluorescence staining in Ishikawa cells were monitored in the presence or absence of TGF-β1 (10 ng/mL) and ISL (10 μM). Green color represents the staining of E-cadherin (**f**) or N-cadherin (**g**). Blue color represents nuclear DNA staining by DAPI (600× magnification). The corrected total cell fluorescence (CTCF) of E-cadherin (**d**) and N-cadherin (**e**) were quantified by ImageJ software. The quantitative values were expressed as the mean ± SD (*n* = 3). * *p* < 0.05, ** *p* < 0.01 versus the vehicle control. # *p* < 0.05, versus the TGF-β1 alone group.

The above results indicate that ISL has a superior migration inhibitory effect on HEC-1A and Ishikawa cells. Next, we wanted to explore whether ISL exerts similar effect on EMT. First, we investigated the effect of TGF-β1 (10 ng/mL) treatment to HEC-1A and Ishikawa cells on expression EMT-related proteins. The results of western blotting showed that treatment of HEC-1A cells (*p* < 0.05, Figure 3a–c) with TGF-β1 for 48 h significantly increased N-cadherin protein expression, while reduced the expression of E-cadherin compared with the control group. Similarly, TGF-β1 significantly reduced the expression of E-cadherin in Ishikawa cells (*p* < 0.05, Figure 4a–c), but the increase in N-cadherin expression failed to reach statistical significance. Simultaneously, using immunofluorescence staining and measuring the level of corrected total cell fluorescence (CTCF) showed similar findings where treatment of HEC-1A (Figure 3d–g) and Ishikawa cells (Figure 4d–g) with TGF-β1 for 48 h significantly increased N-cadherin protein expression, while reduced the expression of E-cadherin compared to the control group (*p* < 0.05). 

**Figure 5:**
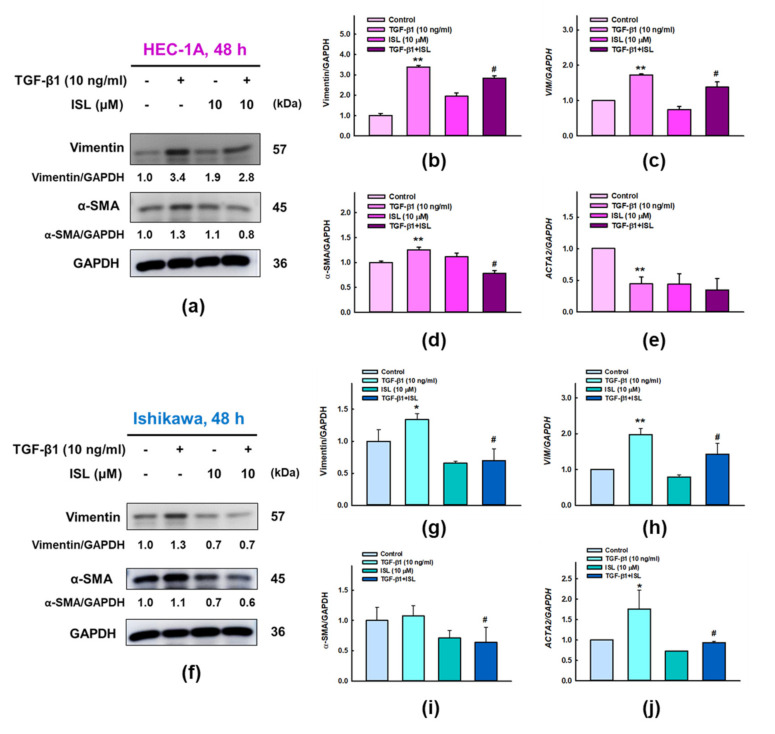
ISL reversed expression of TGF-β1-induced epithelial-mesenchymal transition (EMT) related markers in human endometrial cancer cells. HEC-1A (**a**) and Ishikawa (**f**) cells were treated with TGF-β1 (10 ng/mL) and ISL (10 μM) for 48 h. Cell lysates were separated by SDS-PAGE and subjected to western blotting with the anti-vimentin, and anti-α-SMA. Each target protein was normalized to GAPDH and protein quantification was shown in bar graph (**b**,**d**,**g**,**i**). Relative mRNA expression of *VIM* and *ACTA2* were analyzed by RT-PCR (**c**,**e**,**h**,**j**), the mRNA level of GAPDH was used as an internal control. The quantitative values were expressed as the mean ± SD (*n* =3). * *p* < 0.05, ** *p* < 0.01 versus the vehicle control. # *p* < 0.05, versus the TGF-β1 alone group.

In addition, TGF-β1 significantly increased the protein expression of vimentin in HEC-1A (*p* < 0.01, Figure 5a,b) and Ishikawa cells (*p* < 0.05, Figure 5f,g) as compared to control group. Similarly, TGF-β1 significantly increased the protein expression of α-SMA in HEC-1A cells (*p* < 0.01, Figure 5a,d) compared with control group. But the increase in expression of α-SMA in Ishikawa cells wasn’t statistically significant (Figure 5f,i). Moreover, we wanted to investigate whether ISL can also affect the transcriptional activity of these EMT related markers, we performed qPCR analysis and found the mRNA levels of *VIM* (encoding vimentin) and *ACTA2* (encoding α-SMA) were significantly up-regulated in response to TGF-β1 treatment in Ishikawa cells (*p* < 0.05, Figure 5h,j; 1.97-fold and 1.76-fold, respectively). Only *VIM* mRNA expression was significantly up-regulated by TGF-β1 treatment in HEC-1A cells (*p* < 0.01, Figure 5c; 1.74-fold), while *ACTA2* mRNA expression after TGF-β1 treatment was not consistent with the western blotting result (Figure 5e).

Previous results showed the stimulatory effect of TGF-β1 on EMT related markers in endometrial cancer cells both on protein and gene expression level. Then, we examined whether ISL treatment is able to inhibit these stimulatory effects. The results of western blotting show that 10 μM ISL significantly reversed the increased protein expression of N-cadherin in HEC-1A cells (*p* < 0.05, Figure 3c) and upregulated the protein expression of E-cadherin in Ishikawa cells (*p* < 0.05, Figure 4b), while the protein expression of E-cadherin after ISL treatment showed an increasing trend in HEC-1A cells (Figure 3b), and the protein expression of N-cadherin after ISL treatment showed a downward trend in Ishikawa cells (Figure 4c) without statistical significance. The results of immunofluorescence staining, and subsequently quantifying the level of CTCF, showed that 10 μM ISL significantly reduced the protein expression of N-cadherin (*p* < 0.05, Figure 3e,g), and significantly increased the protein expression of E-cadherin (*p* < 0.05, Figure 3d,f) reversing the stimulatory effect induced by TGF-β1 co-treatment in HEC-1A cells. Similarly, 10 μM ISL also significantly reduced the protein expression of N-cadherin (*p* < 0.05, Figure 4e,g), and significantly increased the protein expression of E-cadherin (*p* < 0.05, Figure 4d,f) reversing the stimulatory effect induced by TGF-β1 co-treatment in Ishikawa cells. Moreover, 10 μM ISL significantly decreased TGF-β1 induced vimentin and α-SMA protein expression (*p* < 0.05) in HEC-1A (Figure 5a,b,d) and Ishikawa (Figure 5f,g,i) cells. Similar results were found at the RNA level, where 10 μM ISL significantly decreased TGF-β1 induced mRNA expression of *VIM* and *ACTA2* (*p* < 0.05) in HEC-1A (Figure 5c) and Ishikawa cells (Figure 5h,j), except for *ACTA2* in HEC-1A where reduction didn’t reach statistical significance (Figure 5e). Collectively, these results demonstrated potential effect of ISL in reversing TGF-β1 induced EMT process towards epithelial-like phenotype in both HEC-1A and Ishikawa cells.

### 2.4. Effect of ISL on Expression of TGFβ/Smad Signaling Pathway Related Markers in Human Endometrial Cancer Cells 

**Figure 6:**
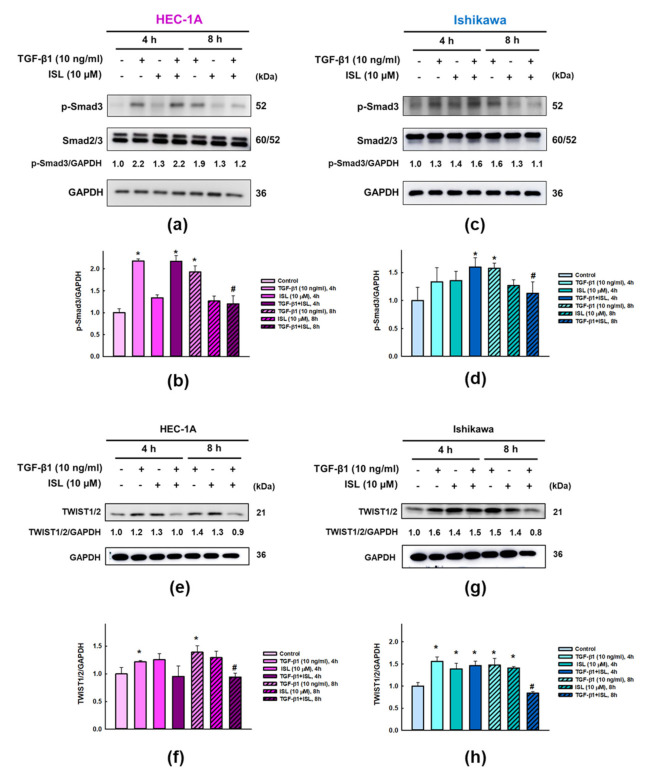
Effect of ISL on expression of TGFβ-/Smad Signaling Pathway related markers in human Endometrial Cancer Cells. HEC-1A (**a**,**e**) and Ishikawa (**c**,**g**) cells were treated with TGF-β1 (10 ng/mL) and ISL (10 μM) for 4 h and 8 h. Cell lysates were separated by SDS-PAGE and detected by western blotting with the anti-p-Smad3, Smad2/3, and TWIST1/2. Each target protein was normalized to GAPDH and protein quantification was shown in bar graph (**b**,**d**,**f**–**h**). The values were expressed as the mean ± SD (*n* = 3). * *p* < 0.05, versus the vehicle control. # *p* < 0.05, versus the TGF-β1 group.

Previous studies showed that TGF-β1 induced EMT via SMAD-dependent or SMAD-independent signaling pathway [32]. In order to identify whether ISL exhibited aforementioned effects through TGF-β1-induced Smad pathway in HEC-1A and Ishikawa cells, western blot analysis was performed. The results showed that treatment of HEC-1A and Ishikawa cells with TGF-β1 for 4 h and 8 h significantly increased the phosphorylation of Smad3 (p-Smad3) protein expression (*p* < 0.05), however, ISL co-treatment significantly decreased p-Smad3 protein expression in HEC-1A (Figure 6a,b) and Ishikawa cells (Figure 6c,d) at 8 h (*p* < 0.05). Subsequently, to explore possible effects of ISL on the expression of transcription factors involved in EMT like TWIST1/2 as a key regulator. The results revealed that treatment of HEC-1A and Ishikawa cells with TGF-β1 for 4 h and 8 h significantly increased the TWIST1/2 protein expression (*p* < 0.05), while this induced TWIST1/2 protein expression by TGF-β1 was significantly downregulated by ISL co-treatment in HEC-1A (Figure 6e,f) and Ishikawa cells (Figure 6g,h) at 8 h (*p* < 0.05). Together, these data indicate that ISL might reverses EMT through a TGF-β1/Smad3/TWIST1/2 dependent signaling pathway in human endometrial cancer cells.

### 2.5. ISL Suppresses Peritoneal Dissemination In Vivo

**Figure 7:**
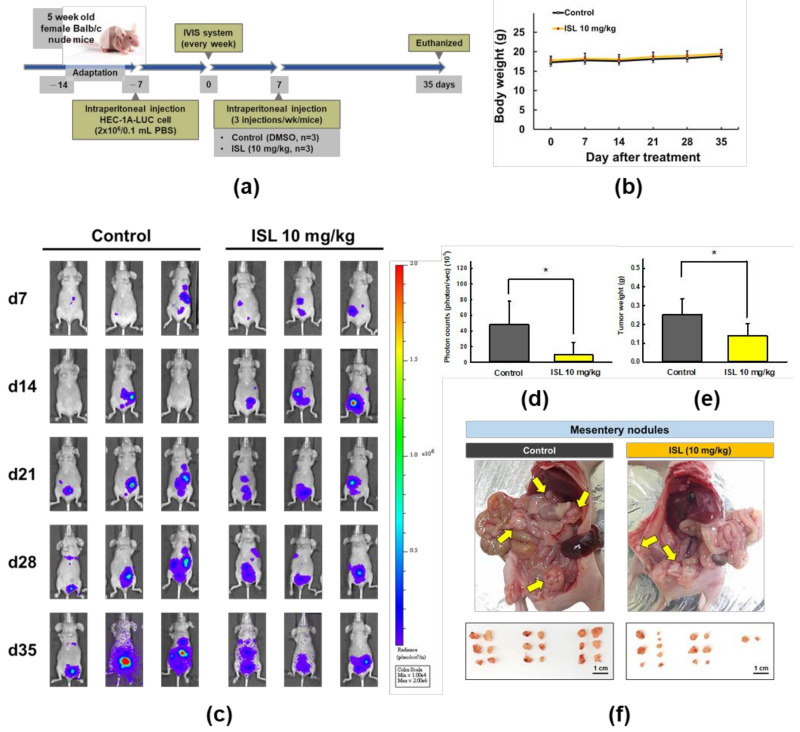
The effect of ISL on peritoneal dissemination in the HEC-1A-LUC mouse xenograft model. Tumors were implanted into mice via intraperitoneal injection of HEC-1A-LUC cells (2 × 10^6^ cells/100 μL PBS), and the mice were injected intraperitoneally with ISL (10 mg/kg) or control (DMSO) every 2–3 days for 28 days. (**a**) Schematic representation of the experimental plan. (**b**) Body weight of nude mice. (**c**) Total flux from luciferase imaging on day 7, 14, 28, and 35 after heterotopic tumor cell injection. (**d**) All luciferase images were normalized to the same photon saturation scale. (**e**) The weight of mesentery nodules and (**f**) nodule distribution state in abdominal cavity of nude mice. Data were expressed as the mean ± SD (*n* = 3). * *p* < 0.05 versus the control.

Next, we examined the effect of ISL on a xenograft animal model (Figure 7a). During the experiment, body weight of the nude mice was measured once a week. The results show that there was no significant difference in body weight between the control group and the ISL group (Figure 7b). In addition, after heterotopic tumor cell injection into mice, tumor luminescence was observed in all mice by In vivo imaging system (IVIS), the total flux from luciferase imaging was tracked and we found that the signal in the ISL group is statistically lower than that in the control group on day 35 (*p* < 0.05, Figure 7c,d). Furthermore, following animals’ euthanization, we found that ISL significantly decreased tumor weight (*p* < 0.05, Figure 7e) and reduced the number of nodules in the mesentery of the ISL group (Figure 7f). In addition, the number of nodule distribution in abdominal cavity of nude mice in the ISL group was lower than in the control group (Figure 7f). 

**Figure 8:**
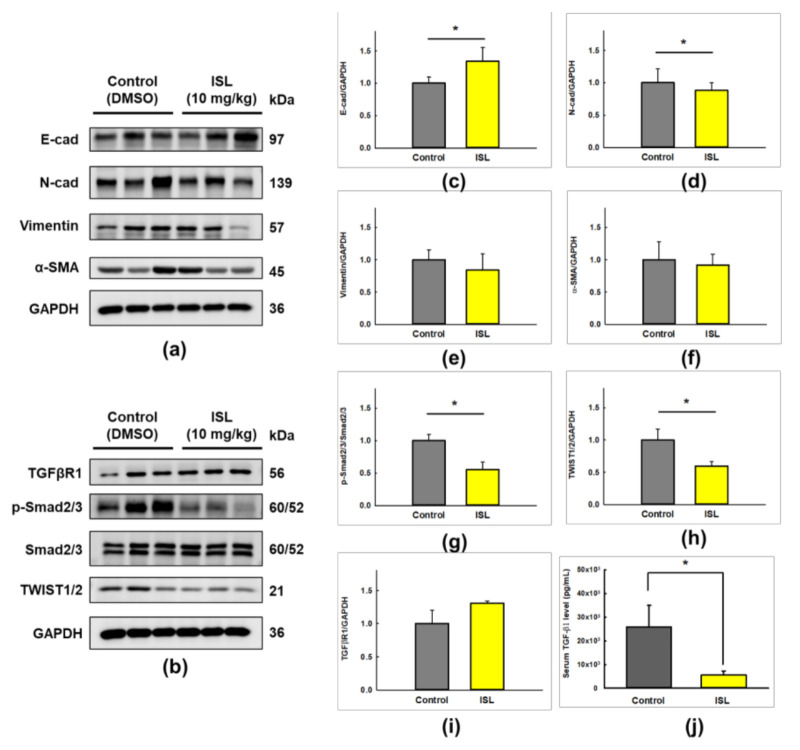
The effect of ISL on EMT related markers expression in the HEC-1A-LUC mouse xenograft model. Tumors were implanted into mice by intraperitoneal injection of HEC-1A-LUC cells (2 × 10^6^ cells/100 μL PBS), and the mice were injected intraperitoneally with ISL (10 mg/kg) or control (DMSO) every 2–3 days for 28 days. The protein expression of E-cadherin, N-cadherin, vimentin, α-SMA, TGFβRI, p-Smad2/3, Smad2/3, and TWIST1/2 in whole cell extracts from tumor tissues were determined by western blotting (**a**,**b**). Each target protein was normalized to GAPDH and protein quantification was shown in bar graph (**c**–**i**). The serum level of TGF-β1 was analyzed by a commercial ELISA kit (**j**). Data were expressed as the mean ± SD (*n* = 3). * *p* < 0.05 versus the control.

After the tumor was removed from the abdominal cavity, expression of EMT-related proteins was analyzed (Figure 8a,b). The results showed that ISL decreased the expression levels of N-cadherin (*p* < 0.05, Figure 8d), vimentin (Figure 8e), α-SMA (Figure 8f), p-Smad2/3 (*p* < 0.05, Figure 8g), and TWIST1/2 (*p* < 0.05, Figure 8h), while increased E-cadherin (*p* < 0.05, Figure 8c) and TGFβRI (Figure 8i) expression levels compared with those of the control group. In addition, we found that the total serum level of TGF-β1 in ISL group was markedly lower than the control group (mean, 5598 pg/mL vs. 25916 pg/mL; *p* < 0.05, Figure 8j). Collectively, these results demonstrated that ISL inhibited the tumor metastasis via up-regulating the E-cadherin and down-regulating the N-cadherin, p-Smad2/3, and TWIST1/2 protein expression in the xenograft animal model. 

## 3. Discussion

To the best of our knowledge, this is the first study to explore the anti-metastasis effect of ISL on endometrial carcinoma in vitro and in vivo. Both preclinical studies demonstrate that ISL could inhibit TGF-β/Smad-induced endometrial cancer cell migration through reducing p-Smad2/3 and TWIST1/2 expression, subsequently decreasing N-cadherin, vimentin, α-SMA while increasing E-cadherin protein expression.

In general, the grading of the neoplasm and depth of myometrial invasion are good indicators of the spread of the disease. In this study, we used HEC-1A and Ishikawa cells as in vitro models, which are originally established from a moderately differentiated (G2) and well-differentiated (G1) endometrial adenocarcinoma, respectively. Although they are all classified as low metastatic endometrial cancer cells, endometrial carcinoma patients still have about 3% (in grade 1) and 9% (in grade 2) of pelvic dissemination and 2% (in grade 1) and 5% (in grade 2) of para-aortic dissemination, respectively [33]. Thus, we used HEC-1A cells transfected with luciferase as dissemination pattern to imitate the advanced stage of endometrial carcinoma in patients in our animal model.

Metastasis is the major cause of death in patient with endometrial cancer. During the process of tumor metastasis, epithelial–mesenchymal transition (EMT) plays an essential role in cancer progression and thus is attracting a high degree of attention [34]. Briefly, EMT serves tumor cells a transition from an epithelial morphology to a motile mesenchymal (migratory and invasive) phenotype, then promote tumor cells to permeate the basal lamina barrier and invade the neighboring tissue. The mechanism of EMT involves downregulated epithelial cell-specific adhesion proteins (E-cadherin) and upregulated mesenchymal proteins (N-cadherin, vimentin, α-SMA and fibronectin) [35]. In previous study, a meta-analysis showed that patients with endometrial cancer have lower expression of E-cadherin than normal people [36]. Moreover, Lei et al also demonstrated that HEC-1-A cells express a low level of E-cadherin and high level of vimentin in the control group [37].Interestingly, these phenotypes were reversed by overexpression of dominant-negative TGF-β receptor type II, which compete with the natural receptors for TGF-β ligand, thus they speculated that the EMT was induced by autocrine TGF-β signaling.

TGF-β signaling is known as the main altered pathway at the core of the molecular network related to the acquisition of an aggressive phenotype in endometrial carcinomas [38]. Particularly, several studies demonstrated that TGF-β enhances the later phases of tumor progression and the metastasis in endometrial cancer [37], partly due to its promotion of EMT [39]. Sun Y et al found that TGF-β1 significantly downregulated E-cadherin expression and upregulated fibronectin, vimentin, and α-SMA expression in MDA-MB-231 cells, these expressions were significantly reversed by resveratrol treatment [40]. Similar results were observed in our present study using HEC-1-A and Ishikawa cells, where TGF-β1 treatment induced a low expression of E-cadherin and high expression of N-cadherin, vimentin, and α-SMA compared to control group, highlighting the role of TGF-β in induction of EMT in endometrial cancer. Moreover, we investigated changes in the mRNA level following treatments as well which was correlated with changes in protein level, except for *ATCA2* mRNA level which was inconsistent with an alpha-SMA protein level after TGF-beta treatment in HEC-1A cells. that might be attributed to a posttranslational modification in protein level beyond transcription or because mRNA is usually prone to degradation.

TGF-β1 was shown to induce both endometrial epithelial and stromal cells, to modulate cells adhesion and differentiation, which eventually leads to metastasis. Muinelo-Romay et al showed that TGF-β1 could promote an invasive phenotype in HEC-1A and RL95-2 cells by inducing EMT signaling [38]. Notably, Xiong et al found that 10 ng/mL of TGF-β1 could induce type II endometrial cancer cell migration [41]. In addition, Feng et al demonstrated that TGF-β-induced morphological change in human lung carcinoma epithelial cells A549 through decreased expression of E-cadherin and induced N-cadherin expression, and eventually lead to migration and invasion [42]. Similar results were obtained in the present study, our results showed that 10 ng/mL of TGF-β1induced morphological changes and the migratory capacity in endometrial cancer cells.

TGF-β Type I receptor (TβRI) and Type II receptor (TβRII) exist on the cell surface in the form of monomers instead of homodimers in the absence of TGF-β. When TGFβ binds directly to the constitutively active TβRII, it triggers the recruitment of the TβRI and activated receptor complexes are formed to activate Smad pathway. The Smad-dependent pathway is the most classic TGF-β signaling pathway, which involves phosphorylation and activation of SMAD2/3 and subsequently interacts with SMAD4 to form a complex that regulates target gene transcription through the interaction with transcriptional cofactors, e.g., SNAIL, ZEB1/2, and TWIST1/2 [32]. On the other hand, TGFβ signaling can also be mediated through Smad independent pathways, including MAP kinase and Akt pathways, thereby stimulating the cell migration. In previous study, Ji et al found that TGF-β binding to its receptor with increased p-Smad2 protein expression, lead to colorectal cancer metastasis [43]. Also, Xiong et al found that the expression of p-Smad2 and p-Smad3 were significantly enhanced upon TGF-β1 exposure in endometrial cancer KLE and HEC-50 cells [41]. Similarly, our results showed that TGF-β1 could increase the protein expression of p-Smad3 and TWIST1/2 in both HEC-1A and Ishikawa cells at 8 h.

ISL is a flavonoid that showed anti-tumor effects in variety of cancer cells. In our previous study, we showed that ISL at 25 μM induced endometrial cancer cell apoptosis, cell cycle arrest, and autophagy, thereby inhibiting endometrial cancer cell growth [29]. Our current study confirmed that ISL causes a significant loss of viability at 10 uM in endometrial cancer cell lines (HEC-1A, Ishikawa, and RL95-2), but it does not induce apoptosis-related protein expression at this dose in our previous studies [29]. In addition, the initial number of cells in each group is the same in the migration test, so that the influence of dead cells can be ruled out. In view of this, we believe that ISL does cause partially endometrial cancer cell death at this dose, but it is speculated that the main effect is through the inhibition of the EMT pathway. By the way, 10 μM of ISL significantly reduced the growth of cancer cells, but not T HESCs normal endometrial stromal cells, highlighting beneficial selective growth inhibitory effect on cancer cells.

Several studies showed that ISL can inhibit migration and invasion of breast cancer cells [44,45,46]. Our study also showed that 10 μM of ISL significantly abrogated TGF-β1-induced migration capability of HEC-1A and Ishikawa cells. In addition, Li et al previously demonstrated that ISL diminished mesangial matrix accumulation after exposure of high glucose, through suppressing induction effect of TGF-β RI/II and their downstream SMAD signaling, [47]. Similarly, our study showed that 10 μM of ISL reduced TGF-β1-induced expression of N-cadherin, vimentin, and p-Smad3, while increased E-cadherin expression, and finally inhibited the migration of endometrial cancer cells.

To confirm the in vitro findings of anti-metastatic effect of ISL in endometrial cancer cells, we utilized in vivo xenograft animal model of endometrial cancer. Based on the results before the experiment (Figure S2), we believe that an ISL of 10 mg/kg is probably effective. As expected, results showed that ISL inhibited the number of nodule distribution in abdominal cavity of nude mice, which indicating that ISL reduce endometrial cancer migration. Similarly, several studies demonstrated that ISL can inhibit migration and invasion in variety of in vivo cancer models, such as breast cancer and ovary cancer. Wang et al found that ISL inhibited tumor growth and angiogenesis in MDA-MB-231 breast cancer xenografts [45]. Also, Zheng et al demonstrated that ISL inhibits breast cancer metastasis by preventing anoikis resistance, essential prerequisite for metastasis, as well as migration and invasion [46]. In addition, Chen et al shown that ISL prolong the survival of ovary-tumor-bearing mice by blocking EMT [48].

Considering the role TGF-β plays during tumor progression and that its level increases within the tumor microenvironment [49] therefore inhibiting its synthesis may exert beneficial effect. Chen et al revealed that the level of TGF-β was much lower in ISL-treated SKOV3 cells than untreated ones using EMT expression array, which implies ISL potential to reduce the level of TGF-β [48]. Additionally, autocrine fashion of TGF-β signaling can facilitate cell survival and tumor progression, probably pending its receptor level. As Lei et al pointed out, abnormal expression of dominant-negative TGF-β type II receptor significantly repressed the number of metastatic nodules and the metastasis colonies on the lung in a mouse xenograft model of endometrial cancer HEC-1A cells [37]. These results suggest that the abrogation of TGF-β signaling may limit the metastatic potential of HEC-1-A cells in vivo [37]. In our in vivo study, the serum level of TGF-β was remarkably down-regulated after ISL treatment, while the TGF-β typeⅠreceptor was not significantly changed between the two groups, thus we speculate that 10 mg/kg of ISL has the potential to reduce TGF-β levels in endometrial cancer, but this dose may not be enough to affect the expression level of its receptor. Although our study didn’t confirm the association between TGF-β receptor inhibitor and the migration of endometrial cancer cells in vivo, the results showed that ISL reduced the expression of TGFβ serum levels, expression levels of p-Smad2 and TWIST1/2 as well as downstream target markers in vivo, thus we propose that ISL may reduce HEC-1A cells migration via TGFβ signaling.

In the other hand, Nakamura et al. found that hepatocyte growth factor (HGF) secreted by ovarian cancer cells enhanced the percentage of the parietal peritoneum invasion site and increased ascites formation in A2780 injected mice, which indicating that HGF promoted ovarian cancer cell dissemination. Besides, the peritoneum with cancer cell invasion showed weak E-cadherin and cytokeratin expression and strong N-cadherin expression [50]. In concordance with this work, our study has shown that ISL suppressed the growth of a xenograft tumor and reduced peritoneal dissemination, as well as increased the expression of E-cadherin and decreased N-cadherin protein expression in vivo.

Before our study, there was no research evidence in public domain to support the effect of ISL on the metastasis of endometrial cancer cells. Accordingly, our findings in the present study may encourage more research to highlight the potential effect of ISL against endometrial cancer metastasis and investigate the underlying molecular mechanisms.

## 4. Materials and Methods

### 4.1. Cell Culture

The human endometrial cancer cell lines HEC-1A and RL95-2 were purchased from the Food Industry Research and Development Institute (FIRDI; Hsinchu, Taiwan, China) and Culture Collection and Research Center (CCRC; Hsinchu, Taiwan, China). The human endometrial adenocarcinoma cell line Ishikawa was purchased from the European Collection of Authenticated Cell Culture (ECACC; Salisbury, UK). Telomerase-immortalized human endometrial stromal cells (T HESCs) were purchased from the American Type Culture Collection (ATCC; Manassas, VA, USA). HEC-1A cells were cultured in McCoy’s 5A (Sigma-Aldrich, St. Louis, MO, USA). RL95-2 and T HESCs cells were cultured in DMEM/Ham’s F-12 (Caisson Labs, Smithfield, UT, USA), and Ishikawa cells were cultured in MEM (Caisson Labs, Smithfield, UT, USA). Most cells were maintained in medium containing 10% fetal bovine serum (FBS; GIBCO, Grand Island, NY, USA), only Ishikawa cells were grown in medium supplemented with 5% FBS, all of them were incubated at 37 °C with 5% CO_2_. Cell culture medium was collected and checked for mycoplasma using the EZ-PCR-Mycoplasma Test Kit (Biological Industries, Cromwell, CT, USA; Figure S3).

### 4.2. Preparation of Isoliquiritigenin and Transforming Growth Factor-β1

Isoliquiritigenin (ISL; C_15_H_12_O_4_; CAS number: 961-29-5; purity ≥ 98%; Sigma-Aldrich, St. Louis, MO, USA) is a yellow powder insoluble in water with a molecular weight of 256.25 kDa. The powder was dissolved in dimethyl sulphoxide (DMSO; Sigma-Aldrich, St. Louis, MO, USA) to prepare a stock solution of 100 mM and stored at −20 °C until use. The carrier solvent (0.1% DMSO) was added to the control group. Recombinant human transforming growth factor-β (TGF-β1; CAS number: 100–21; purity ≥ 98%; Peprotech, Rocky Hill, NJ, USA) derived from HEK293 cells is a 25.0 kDa protein. The powder was dissolved in citrate acid (Sigma-Aldrich, St. Louis, MO, USA) containing 0.1% BSA to prepare a stock solution of 100 mg/mL and stored at −20 °C until use. The carrier solvent (citrate acid + 0.1% BSA) was added to the control group.

### 4.3. Cell Viability Assay

Effect of treatment with ISL on the cell viability was analyzed by the (3-(4,5-Dimethylthiazol-2-yl)-2,5-diphenyltetrazolium bromide) (MTT) assay. Cells were seeded in 96-well plates (3 × 10^3^ cells per well) and cultured for 24 h and then treated with various dosages of ISL in fresh medium containing 1% serum. At the end of treatment, the medium was replaced by medium containing 1% serum with 0.5 mg/mL MTT (Sigma-Aldrich, St. Louis, MO, USA). Following a 3-h incubation, the MTT solution was removed and replaced by DMSO. Absorbance was measured at 570 nm with reference wavelength of 630 nm using an ELISA reader (BioTek, Winooski, VT, USA).

### 4.4. Wound-Healing Assay

Cells (3 × 10^5^ cells/mL) were seeded in 24-well plates, cultured in an incubator under 5% CO_2_ and at 37 °C for 24 h. Sterile pipette tips were used to draw a line in the middle of each well to form wounds of similar size into the monolayer. The monolayer was washed twice with PBS, and the cells were incubated for 48 h after ISL (1, 5, and 10 μM) or 10 ng/mL of TGF-β1 treatment. Wounded area was observed and photographically recorded using a microscope. The wounded areas were quantified by wound width using the Image J software (National Institutes of Health, Bethesda, MD, USA).

### 4.5. Transwell Migration Assay

The migration capability of endometrial cancer cells was assessed using Transwell^®^ cell culture inserts from Corning (Corning, NY, USA). In brief, 3 × 10^4^ cells were seeded onto the upper chamber in 300 µL of serum-free medium containing ISL (1, 5, and 10 μM) or 10 ng/mL of TGF-β1. The cell migration ability at 48 h was then assessed. Cells that migrated through the basement membrane filter were fixed with 100% methanol for 10 min and stained with Giemsa stain (Sigma-Aldrich, St. Louis, MO, USA). Non-migrated cells on the upper surface of filter were gently scrubbed with cotton. The lower face of the membrane was examined. The crystal violet was extracted with 10% acetic acid, and its absorbance was determined at 600 nm and photographed under a microscope.

### 4.6. Immunofluorescence Analysis

Cells were cultured on sterile glass coverslips and treated with 10 μM ISL or 10 ng/mL of TGF-β1 for 48 h. Cells were then fixed with 4% formaldehyde at room temperature for 10 min, permeabilized using 0.1% triton X-100 (Sigma-Aldrich, St. Louis, MO, USA)/PBS for 15 min, and blocked in 5% bovine serum albumin (BSA; BioShop, Burlington, ON, Canada) buffer in 1X TBST. Cells were incubated with anti-E-cadherin (Santa Cruz Biotechnology, Dallas, TX, USA) and anti-N-cadherin (GeneTex, Irvine, CA, USA) primary antibodies for 1 h at room temperature, washed and then incubated with secondary antibodies (Alexa Fluor^®^ 488 dye; Life Technologies, Gaithersburg, MD, USA) for 45 min at room temperature, and then washed four times with 1X TBST for 20 min. ProLong^®^ Gold Antifade Mountant (Thermo Fisher Scientific, Waltham, MA, USA) was added to the cells and coverslips were mounted on glass slides. Fluorescent images were taken under an inverted fluorescence microscope (Leica, Wetzlar, Germany). The level of corrected total cell fluorescence (CTCF) was calculated using the ImageJ software (National Institutes of Health, Bethesda, MD, USA), and the following formula: CTCF = integrated density—(area of selected cell × mean fluorescence of background readings). The quantitative data were derived from the analysis of 5 fields per coverslip, and a total of three coverslips were analyzed.

### 4.7. Western Blot Analysis

Cell lysates were prepared in ice-cold lysis buffer containing a protease (Roche, Basel, Switzerland) and/or phosphatase inhibitor cocktail (Roche). 20 μg of proteins were boiled for 5 min and then subjected to SDS-PAGE gels. The proteins were transferred to a PVDF membrane, 0.45 µm, for 150 min at 300 mA. The membranes were blocked with 5% BSA (BioShop, Burlington, ON, Canada) for 1 h at room temperature, and incubated with anti-α-SMA (GeneTex, Irvine, CA, USA), anti-E-cadherin (GeneTex), and anti-N-cadherin (GeneTex), anti-p-Smad3 (Cell Signaling Technology, Beverley, MA, USA), anti-Smad2/3 (Cell Signaling Technology), anti-vimentin (Cell Signaling Technology), and anti-horseradish peroxidase (HRP)-conjugated glyceraldehyde 3-phosphate dehydrogenase (GAPDH; Proteintech, Rosemont, IL, USA) primary antibodies at 4 °C overnight. The membranes were washed three times with TBST for 30 min (60 rpm), and the corresponding goat anti-rabbit/mouse antibody IgG (Abcam, Cambridge, UK) was added. After 1 h of shaking at room temperature, the membranes were washed three times with TBST and reacted with electrochemiluminescence (ECL; Thermo Fisher Scientific, Waltham, MA, USA) in the dark for 1 min. The chemiluminescence imaging was detected by a Luminescent Image Analyzer Amersham Imager 600 (GE Healthcare, Marlborough, MA, USA). Densitometric estimations were quantified using the Image J software (National Institutes of Health, Bethesda, MD, USA). All of the raw data using western blotting were supplemented in Figure S1.

### 4.8. RNA Isolation, Reverse Transcription and Quantitative Real-Time PCR (qPCR) Analysis

Total RNA from TGF-β1 (10 ng/mL)- or ISL (10 μM)-treated HEC-1A and Ishikawa cells were extracted using TRIzol™ Reagent (Invitrogen, Carlsbad, CA, USA) followed by purification with Direct-zol RNA MiniPrep (Thermo Fisher Scientific, Waltham, MA, USA). cDNA was reverse transcribed from 2 µg total RNA using PrimeScript^®^ RT Reagent Kit (Takara, Kusatsu, Shiga, Japan) according to the manufacturer’s guidelines. mRNA levels of genes of interests were measured with gene-specific primers and Power SYBR Green Master Mix (Thermo Fisher Scientific, Waltham, MA, USA). All samples were analyzed in triplicate. Used qPCR primers are listed in Table 1. Real-time PCR analyses were performed with Applied Biosystems StepOnePlus™ Real-Time PCR System (Thermo Fisher Scientific, Waltham, MA, USA). Amplification of all genes was performed under the following cycling conditions: denaturation at 95 °C for 10 min followed by 40 cycles for 15 s at 95 °C and 30 s at 60 °C. Synthesis of DNA product of the expected size was confirmed by DNA electrophoresis and melting curve analysis. Relative expression levels were normalized to internal control GAPDH and calculated by 2^−∆∆Ct^ method.

### 4.9. Tumor Xenograft in Nude Mice

All animal studies were conducted according to the protocols approved by the Institutional Animal Care and Use Committee (IACUC) of Taipei Medical University (IACUC Approval No. 2015-0447). Five-week-old female nude mice (CAnN.Cg-Foxn1^nu^/CrlNarl; National Laboratory Animal Center, Taipei, Taiwan) were housed on a 12-h light/12-h dark cycle in a pathogen-free environment and allowed food and water ad libitum. After the one-week adaptation period, we implanted tumors into mice by intraperitoneal injection of HEC-1A-LUC cells (2 × 10^6^ suspended in 100 μL of PBS). All of the mice received every 2–3 days an intraperitoneal injection of ISL (10 mg/kg) or vehicle (DMSO) for 28 days after randomly divided into two groups (*n* = 3 per group). The body weight was recorded every week. Mice were injected intraperitoneally with 100 mg/kg luciferin (VivoGlo™ Luciferin In Vivo Grade; Promega, Madison, WI, USA) for 10 min before anesthetized with 2.5 % isoflurane (Attane; Panion & BF Biotech Inc., Taipei, Taiwan) combined with 100% oxygen and detected by a Non-Invasive In Vivo Imaging System (IVIS; Xenogen, Hopkinton, MA, USA). At the end of the experiment, the mice were euthanized with mixture solution (1 mL Zoletil and 0.1 mL rompun in 3.9 mL normal saline), and tumor weight were recorded as well as the total weight of mesentery nodule in the abdominal cavity. Collected samples were used for analyzing the expression of specific proteins in the tumors, including E-cadherin, N-cadherin, α-SMA, TGFβRI (GeneTex), vimentin, p-Smad2/3, Smad2/3, TWIST1/2 (Cell Signalling Technology), and GAPDH (Proteintech, Rosemont, IL, USA).

### 4.10. Enzyme-Linked Immunosorbent Assay (ELISA).

Levels of TGFβ1 in mouse serum were measured using a Mouse TGF-beta 1 DuoSet ELISA kit (Catalog# DY1679-05 from R&D Systems, Minneapolis, MN, USA) according to the manufacturer’s guidelines. Briefly, total TGFβ1 levels were measured in 40 μL of serum by mixing these with 10 μL 1 N HCl, followed by neutralization with 10 μL 1.2 N NaOH/0.5 M HEPES, according to the manufacturer’s sample activation procedure. These samples then were diluted 1:30 in assay diluent for measurement of total TGFβ1. ELISA plates were analyzed by reading absorbance using a microplate reader set to 450 nm with wavelength correction set to 570 nm.

### 4.11. Statistical Analysis

Data were presented as the mean ± standard deviation (SD). Statistical differences between the means were analyzed by one-way analysis of variance (ANOVA) testing using the SigmaPlot, version 12.5 (SoftHome, Taipei, Taiwan, China). Group means were compared using one-way ANOVA and Tukey post hoc test. For comparison of two groups, Student’s t-test was used. The difference between two means was considered statistically significant when *p* < 0.05 and highly significant when *p* < 0.01.

## 5. Conclusions

**Figure 9:**
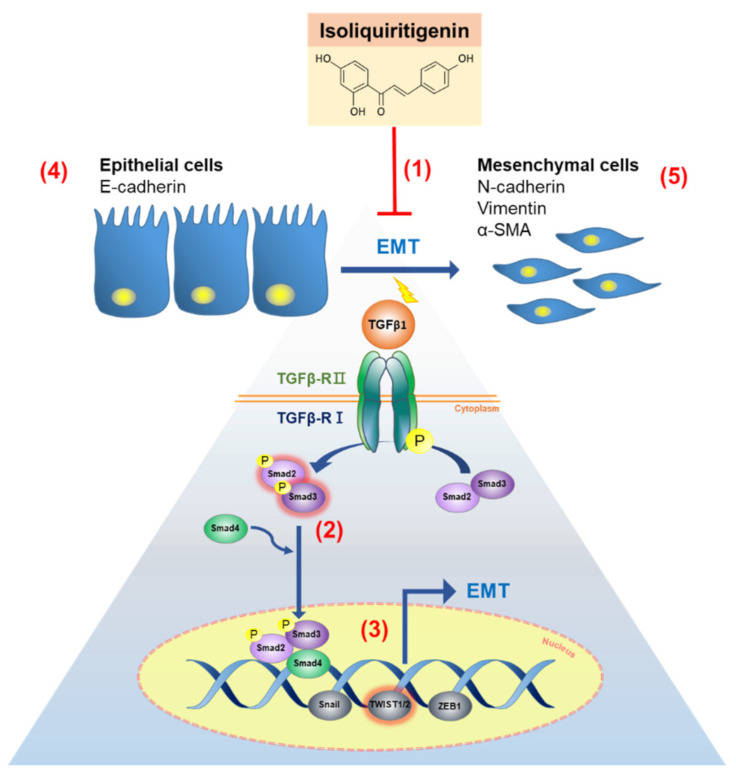
Schematic diagram of ISL effect on TGF-β1 induced epithelial-mesenchymal transition (EMT) in human endometrial cancer. (1) ISL arrests the EMT progress via decreasing cells migration ability. (2,3) ISL reduces p-Smad3 expression and prevents its translocating into the nucleus and subsequent binding with TWIST1/2 (transcription factor) to regulate target gene expression. (4–5) ISL reverses the effect of TGF-β by restoring the loss on the epithelial marker (E-cadherin) and revoking the gain of mesenchymal markers (N-cadherin, vimentin, and α-SMA). Abbreviation: transforming growth factor beta (TGF-β); epithelial–mesenchymal transition (EMT).

The results of this study demonstrated that ISL could reverse TGF-β-induced endometrial cancer cell migration through inhibition of TGF-β/Smad signaling pathway with subsequent increased E-cadherin expression and reduced N-cadherin, vimentin, α-SMA, p-Smad3, and TWIST1/2 expression (Figure 9). In the in vivo experiment, ISL reduced peritoneal dissemination and serum level of TGF-β1, as well as decreased the levels of N-cadherin, p-Smad2/3, TWIST1/2 expression, while increased E-cadherin expression levels. These findings indicate that ISL has a potential therapeutic effect on the treatment of metastatic endometrial cancer.

## Figures and Tables

**Table 1:** Sequences of qPCR primers.

Gene	Forward (5′ to 3′)	Reverse (5′ to 3′)
*VIM*	AGTCCACTGAGTACCGGAGAC	CATTTCACGCATCTGGCGTTC
*ACTA2*	GCTTGTCCAGGAGTTCCGCTCC	GATGCGAAGTGCTGACCCCGC
*GAPDH*	TGCACCACCAACTGCTTAGC	GGCATGGACTGTGGTCATGAG

## Data Availability

Not applicable.

## Supplementary Materials

The following are available online at https://www.mdpi.com/2072-6694/13/6/1236/s1, Figure S1. The raw data of western blotting; Figure S2. The raw data of animal pre-experiment. Figure S3. The raw data of mycoplasma test.
