# Supplementary material for: Isoliquiritigenin Reverses Epithelial-Mesenchymal Transition Through Modulation of the TGF-β/Smad Signaling Pathway in Endometrial Cancer
Source: Cancers (Basel). 2021 Mar 11;13(6):1236. doi: 10.3390/cancers13061236 (PMC8001359; doi:10.3390/cancers13061236)
Supplement: Supplementary file 1 [file cancers-13-01236-s001.pdf]

Isoliquiritigenin Restrains Epithelial-Mesenchymal Transition in Endometrial Cancer Cells via the TGF-β/Smad Signaling Pathways

--Supplementary Figure S1—S3

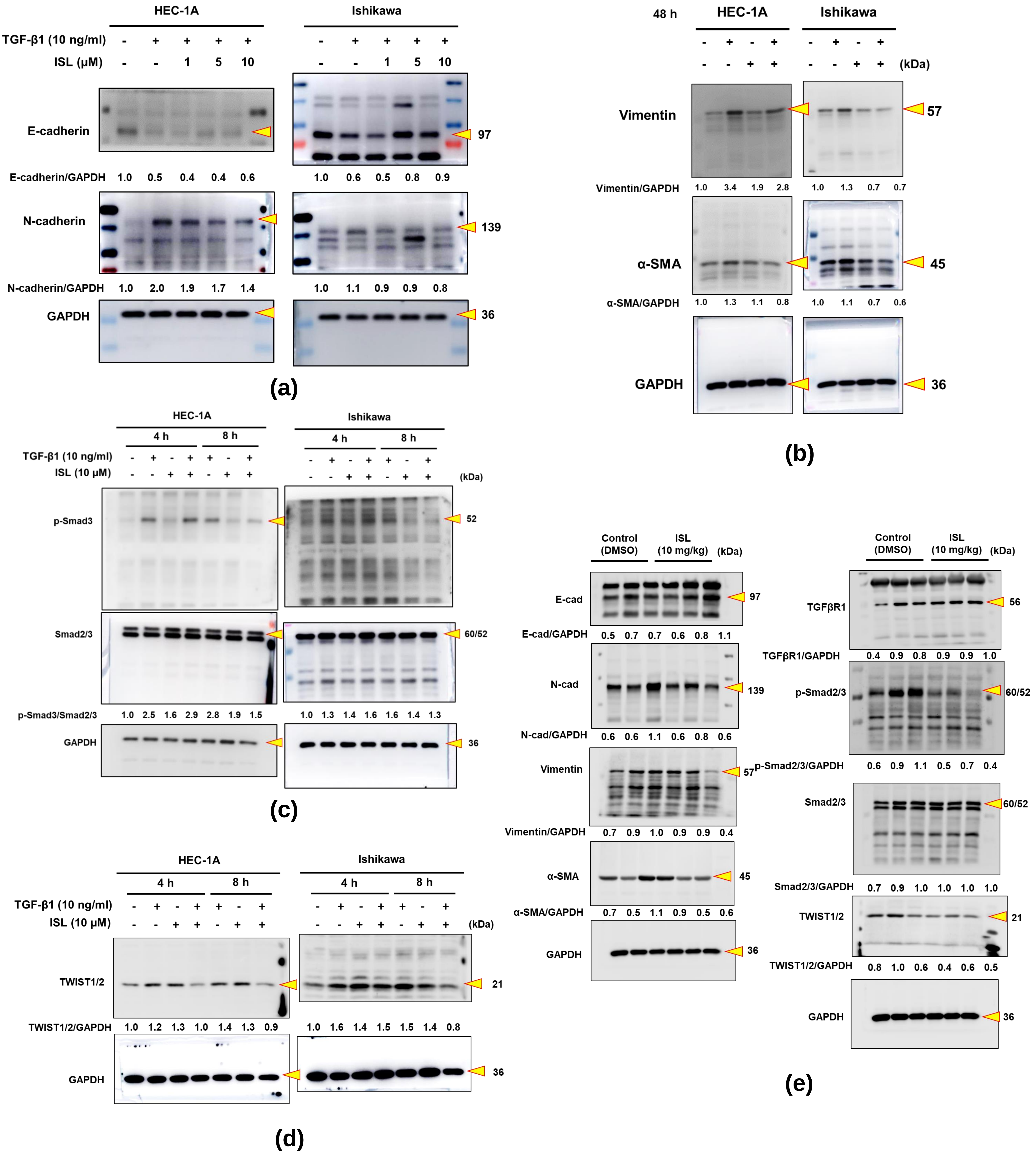

Supplementary Figure S1. The raw data of western blotting.

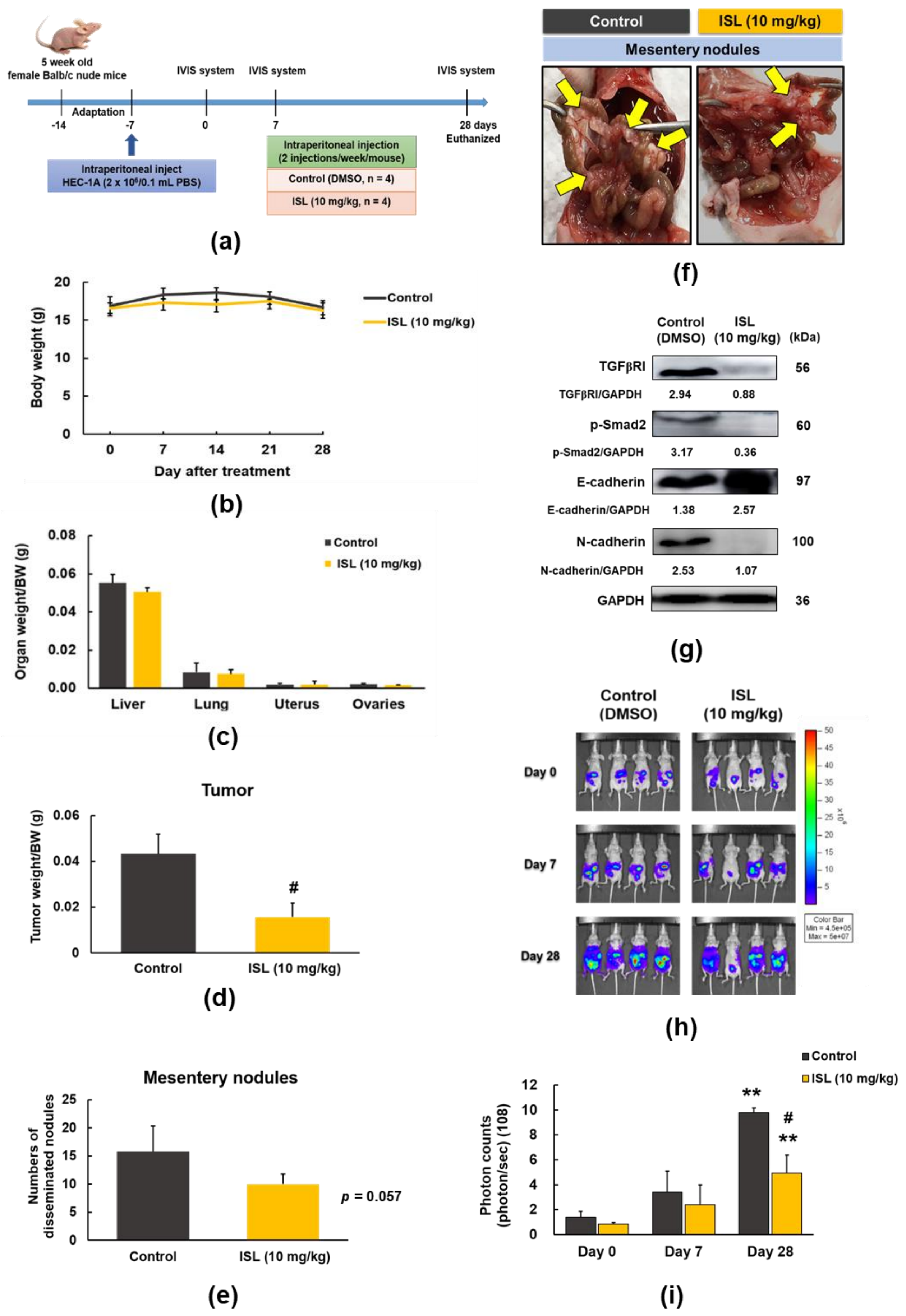

Supplementary Figure S2. The raw data of animal pre-experiment.

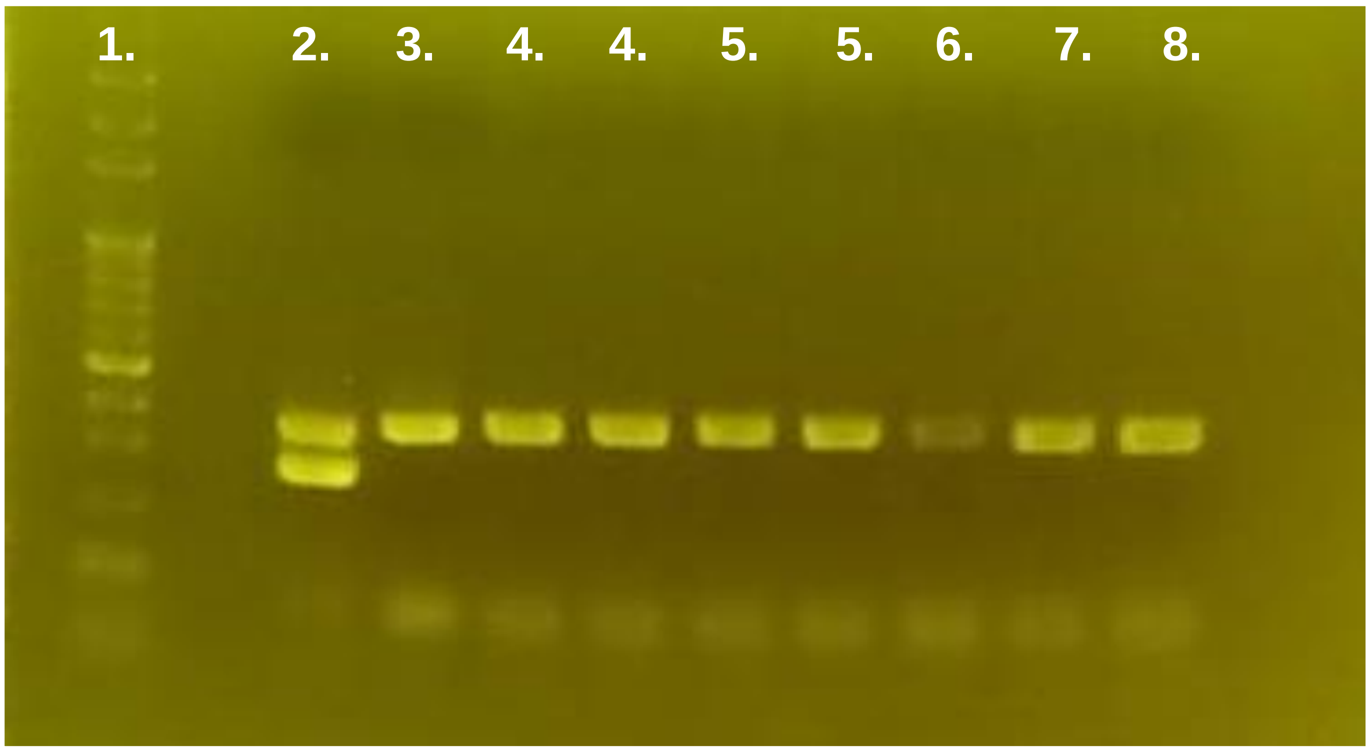

Supplementary Figure S3. The raw data of mycoplasma test. 1. Marker, 2. Positive control, 3. Negative control, 4. HEC-1A cells, 5. Ishikawa cells, 6. ELT-3 cells, 7. HaCaT cells, 8. RIN-m5F cells.
